# Building a successful minimally invasive mitral valve repair program before introducing the robotic approach: The Massachusetts General Hospital experience

**DOI:** 10.3389/fcvm.2023.1113908

**Published:** 2023-03-21

**Authors:** Antonia van Kampen, Guillaume Goudot, Sophie Butte, Dane C. Paneitz, Michael A. Borger, Vinay Badhwar, Thoralf M. Sundt, Nathaniel B. Langer, Serguei Melnitchouk

**Affiliations:** ^1^ Division of Cardiac Surgery, Massachusetts General Hospital, Harvard Medical School, Boston, MA, United States; ^2^ University Clinic for Cardiac Surgery, Leipzig Heart Center, Leipzig, Germany; ^3^ Division of Cardiology, Massachusetts General Hospital, Harvard Medical School, Boston, MA, United States; ^4^ Department of Cardiovascular and Thoracic Surgery, West Virginia University Heart and Vascular Institute, Morgantown, WV, United States

**Keywords:** mitral valve (MV) repair, mitral valve prolapse, minimally invasive cardiac surgery, minimally invasive mitral valve repair, robotic cardiac surgery, robotic mitral valve repair

## Abstract

**Background:**

Patients with mitral valve prolapse (MVP) requiring surgical repair (MVr) are increasingly operated using minimally invasive strategies. Skill acquisition may be facilitated by a dedicated MVr program. We present here our institutional experience in establishing minimally invasive MVr (starting in 2014), laying the foundation to introduce robotic MVr.

**Methods:**

We reviewed all patients that had undergone MVr for MVP *via* sternotomy or mini-thoracotomy between January 2013 and December 2020 at our institution. In addition, all cases of robotic MVr between January 2021 and August 2022 were analyzed. Case complexity, repair techniques, and outcomes are presented for the conventional sternotomy, right mini-thoracotomy and robotic approaches. A subgroup analysis comparing only isolated MVr cases *via* sternotomy vs. right mini-thoracotomy was conducted using propensity score matching.

**Results:**

Between 2013 and 2020, 799 patients were operated for native MVP at our institution, of which 761 (95.2%) received planned MVr (263 [34.6%] via mini-thoracotomy) and 38 (4.8%) received planned MV replacement. With increasing proportions of minimally invasive procedures (2014: 14.8%, 2020: 46.5%), we observed a continuous growth in overall institutional volume of MVP (*n* = 69 in 2013; *n* = 127 in 2020) and markedly improved institutional rates of successful MVr, with 95.4% in 2013 vs. 99.2% in 2020. Over this period, a higher complexity of cases were treated minimally-invasively and increased use of neochord implantation ± limited leaflet resection was observed. Patients operated minimally invasively had longer aortic cross-clamp times (94 vs. 88 min, *p* = 0.001) but shorter ventilation times (4.4 vs. 4.8 h, *p* = 0.002) and hospital stays (5 vs. 6 days, *p* < 0.001) than those operated *via* sternotomy, with no significant differences in other outcome variables. A total of 16 patients underwent robotically assisted MVr with successful repair in all cases.

**Conclusion:**

A focused approach towards minimally invasive MVr has transformed the overall MVr strategy (incision; repair techniques) at our institution, leading to a growth in MVr volume and improved repair rates without significant complications. On this foundation, robotic MVr was first introduced at our institution in 2021 with excellent outcomes. This emphasizes the importance of building a competent team to perform these challenging operations, especially during the initial learning curve.

## Introduction

Cardiac surgery faces the challenge of integrating ongoing innovations, allowing constant progress, while requiring a significant effort to update and train surgeons in technically challenging and unforgiving procedures. Mitral valve (MV) surgery represents an archetype of this challenge, since for this procedure, conventional full sternotomy has been increasingly replaced by minimally invasive and robotic approaches that aim to limit postoperative complications and allow faster patient recovery while maintaining a high level of technical success (1, 2). The literature leaves little room for doubt: minimally invasive MV surgery has become a standard approach with excellent short- and long-term results (3–6). However, the integration of these procedures, still often limited to tertiary care centers, has been slowed down by learning curves, the need for a time-consuming training program, and the risk of a transient increase in postoperative complications (5). Recently, robotically assisted mitral valve surgery has been gaining increasing popularity, due to potentially even smaller incisions and certain advances in surgical exposure.

Massachusetts General Hospital (MGH) established a focused MV surgery innovation program in 2014, dedicated to mitral valve repair (MVr) *via* a minimally invasive approach and the introduction of the robotic approach as further innovation. Previously published studies demonstrated that learning curves for minimally invasive MVr can be overcome safely (4, 7) and adverse events can potentially be reduced by dedicated and standardized teaching procedures (8). We aim to analyze the results of our MVr program over eight years in terms of the number and type of mitral procedures, as well as peri- and postoperative outcomes, and present our experience with introducing the robotic MVr approach.

## Methods

### Patient inclusion and data collection

All patients that had undergone MV surgery at Massachusetts General Hospital between January 2013 and December 2020 were identified *via* interrogation of the Society of Thoracic Surgeons database. In addition, we included all patients that had received robotically assisted MVr at our institution in 2021 and 2022 to compare the initial outcomes of that innovative approach to previous outcomes. All perioperative notes, surgical reports, and imaging findings were reviewed. The planned operative strategy (repair vs. replacement, minimally invasive vs. conventional approach) was obtained from the perioperative notes, and intraoperative complications or changes in strategy were registered as reported by the individual surgeon. Standard preoperative transthoracic and intraoperative transesophageal echocardiography reports were utilized to evaluate MV pathology and procedural success. Successful repair was defined as MVr without requiring intraoperative conversion to MV replacement. The study was approved, and individual informed consent waived by the Institutional Review Board of Massachusetts General Hospital. *Written informed consent was obtained from the individuals* for the publication of any potentially identifiable images or data included in this article.

### Selection criteria for minimally invasive mitral valve repair

Patients referred for MVr undergo routine preoperative transthoracic echocardiography and either coronary angiography or ECG-gated CT angiography for coronary assessment. In addition, CT angiography of the entire aorta and iliac vessels is added if a patient is considered for a minimally invasive approach. Patients are then scheduled to have a full sternotomy approach if additional valve surgery or coronary artery bypass grafting (CABG) is indicated. The most common exclusion criteria for minimally invasive MVr include dilation of the ascending aorta > 45 mm, significant mitral annular calcification, presence of breast implants, significant pectus excavatum, left diaphragm paralysis, and previous right thoracotomy or sternotomy. Patients with low left ventricular ejection fraction (LVEF) or a predicted risk of mortality > 4% (moderate surgical risk category) by the Society of Thoracic Surgeons (STS PROM) risk score are also preferably operated on *via* a full sternotomy, to keep aortic cross-clamp times as short as possible. Furthermore, patients with significant calcifications or soft plaques in the descending aorta are not eligible for minimally invasive MVr at our institution, because of the need for retrograde aortic perfusion. Among our staff, 2 individuals perform minimally invasive MVr, while the other surgeons continue to use the sternotomy approach in all MVP patients referred to their practice.

### Subgroup analysis of patients undergoing isolated mitral valve repair

**Figure 1:**
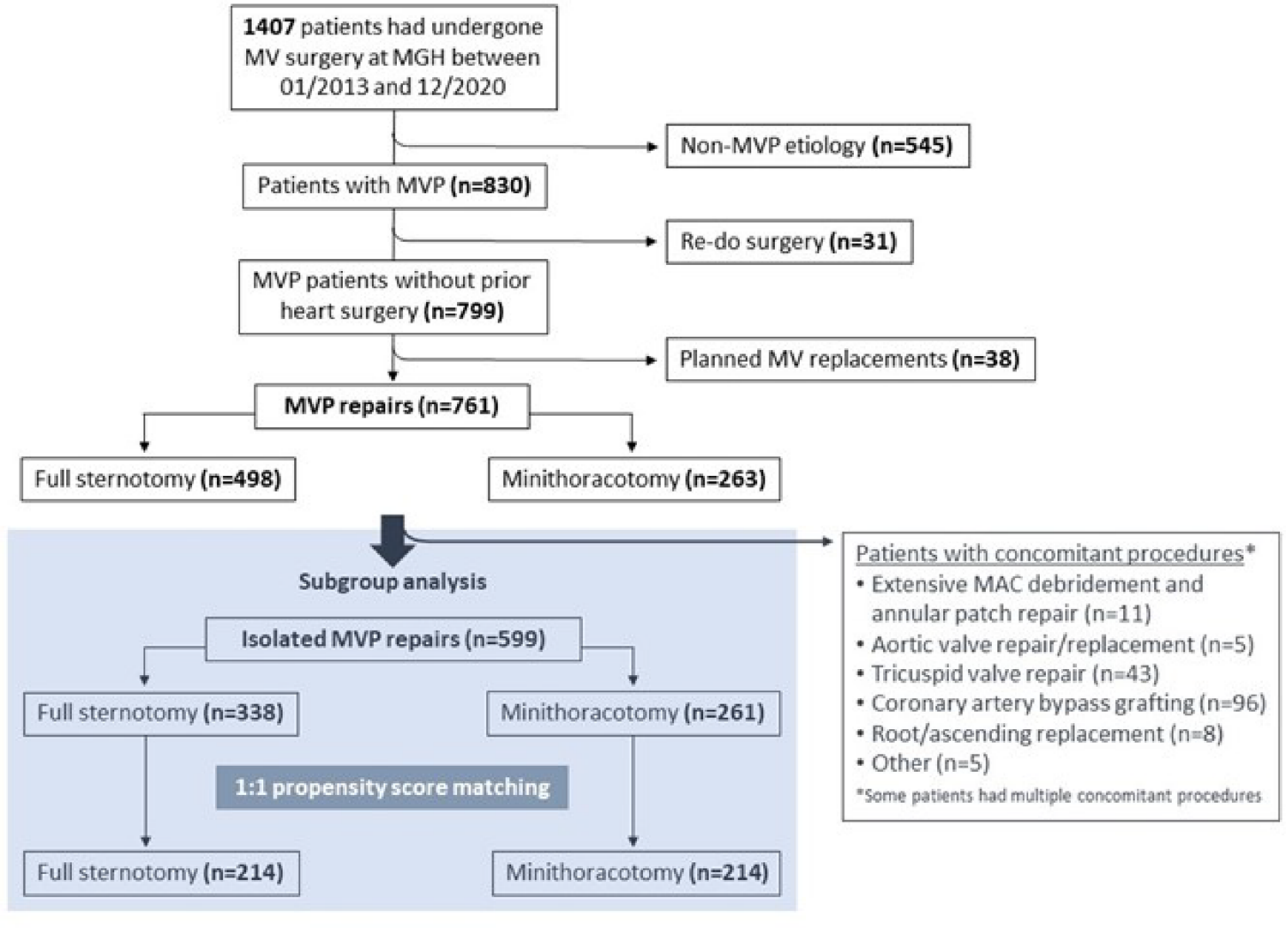
CONSORT diagram of patient selection for minimally invasive vs. sternotomy MVr. MAC = mitral annular calcificiation; MVP = mitral valve prolapse.

To directly compare outcomes of conventional and minimally invasive MVr, patients undergoing isolated MVr were identified from the larger patient cohort excluding those that had major concomitant procedures prohibiting minimally invasive access at our institution i.e., CABG, surgery on aortic, tricuspid, or pulmonary valve, surgery on the ascending aorta or aortic arch, those with acute endocarditis, and those with severe mitral annular calcification requiring debridement and annular patch repair (Figure 1). Non-exclusionary concomitant procedures included closure of an atrial septal defect or a persistent foramen ovale (PFO), the Cox-Maze procedure, and occlusion of the left atrial appendage.

### Establishing robotic surgery for mitral valve prolapse

The extensive expertise gained throughout the years of successfully performing minimally invasive MVr was the essential foundation to launch a robotic MVr program in 2021, with the goal of obtaining similar excellent outcomes without compromising patient safety and facilitating certain steps of the procedure. Due to less restricted mobility and orientation of the surgical instruments during robotic surgery, exposure of and access to the papillary muscles for neochord implantation is superior to standard minimally-invasive approaches with long-shafted one-directional instruments. Despite initially expected longer procedural times, we believe that by introducing the robotic approach, we can further improve patient care and will eventually achieve equal or even shorter procedural times as with the established techniques.

**Figure 2:**
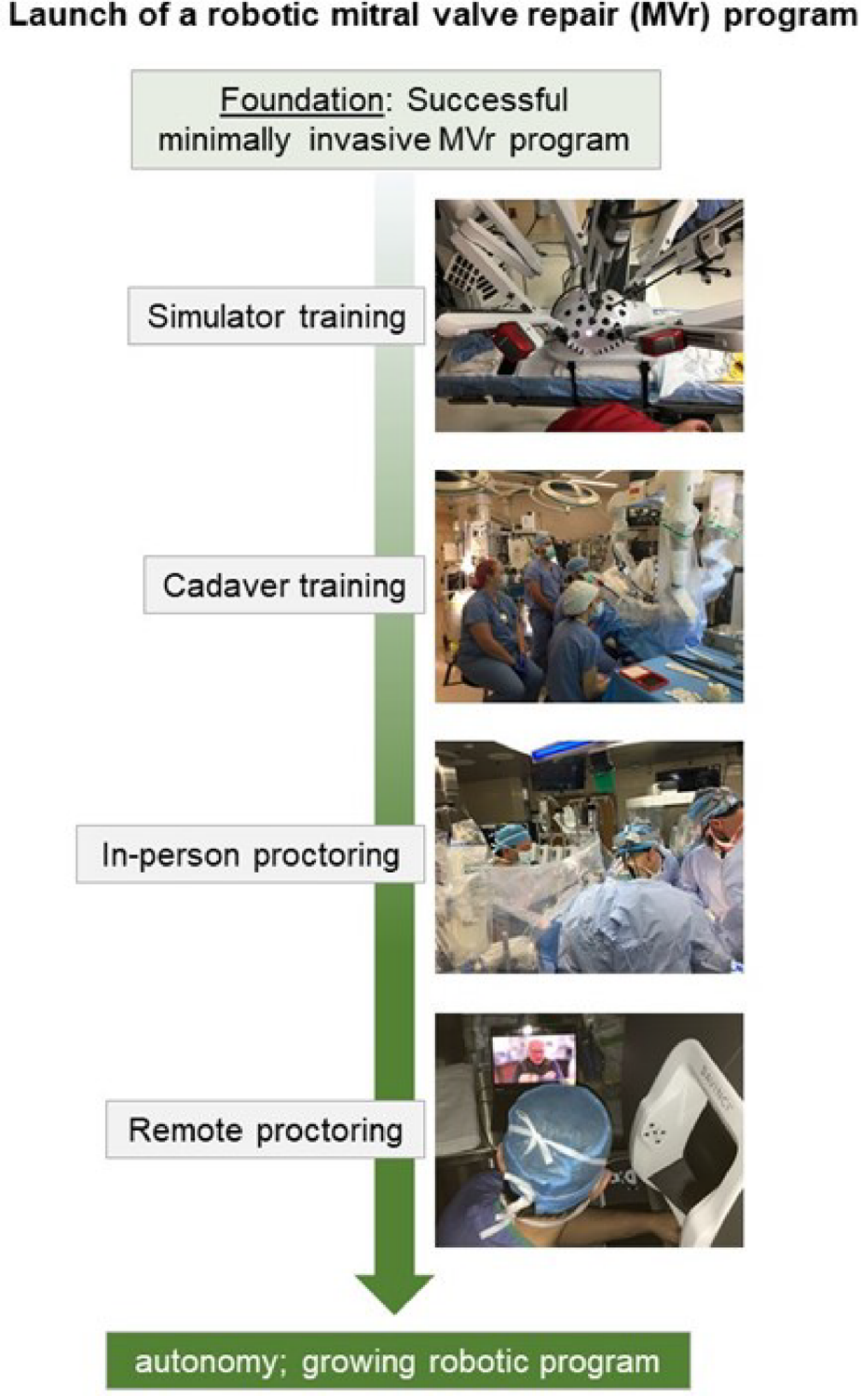
Stepwise approach to launching a robotic mitral valve repair program at Massachusetts general hospital.

At our institution, the most experienced minimally-invasive MV surgeon and team consisting of a co-surgeon, a scrub technician, a perfusionist, and an anesthesiologist, underwent comprehensive training *via* a society-supported formal training program including simulator and cadaver training. In-person proctoring was done for the first 5 cases, and remote proctoring for the sixth, seventh, and eighth cases. This stepwise approach (Figure 2) led to a successful launch of our robotic MVr program (9). For the initial cases included in this analysis, we added selection criteria to the ones mentioned above for minimally invasive surgery: Because we suspected initially prolonged procedural times, we included patients with very low perioperative risk (STS PROM <1%) and focused on pathologies deemed uncomplicated to repair based on preoperative echocardiography. Robotic MVr is currently available on a weekly basis at our institution and offered to all patients eligible for minimally invasive MVr, as described above. All patients that had undergone robotic MVr at our institution until August of 2022 were included in this analysis.

### Statistical analysis

After assessment for normal distribution, continuous variables were expressed as median with interquartile range [25th—75th percentile], and categorical variables as numbers with percentages. Unmatched group comparisons were conducted using the Wilcoxon rank sum test or Fisher's exact test, as appropriate. For the subgroup analysis, propensity scores were calculated using logistic regression. A 1:1 propensity score analysis was conducted using the “nearest neighbor” algorithm, with a caliper setting of 0.1 standard deviations and without replacement. Before matching, multiple imputations was used to compensate for missing data. Standardized mean differences were used to evaluate the balancing of covariates after matching. Variables used to calculate the propensity score were age, sex, Barlow's valve, arterial hypertension, diabetes, peripheral arterial disease, previous stroke, chronic obstructive lung disease, preoperative LVEF, body mass index, and STS PROM.

After matching, continuous variables were reported as median with interquartile range and compared using Wilcoxon signed rank test; categorical variables were reported as numbers with percentages and compared using McNemar's test. Long-term freedom from re-operation and death was examined using Kaplan-Meier method with log-rank test. Data analysis was conducted using R software (R-Studio, version 3.4.1, Boston, MA, United States).

## Results

### Patient cohort

A total of 1407 patients underwent MV surgery *via* full sternotomy or right anterolateral mini-thoracotomy at our institution between January 2013 and December 2020. Of them, 830 patients were operated for mitral valve prolapse (MVP), of which 38 received a planned MV replacement (common reasons were too complex valve anatomy, severe mitral annular calcifications, or patient preference) and 31 were re-operations. Surgical repair of native MVP was thus performed in 761 patients, and those were included for further analysis (Figure 1). Table 1 shows the preoperative characteristics of all 761 patients. In summary, the median age was 64 [55–72] years, and 245 patients (32.2%) were female. Mitral regurgitation was severe in 720 (94.9%), moderate in 37 patients (4.9%), and less than moderate in 4 patients (0.2%) (MVP was not the primary indication for surgery in those latter two groups). Significant mitral annular calcification was present in 38 patients (5%). Moderate or more aortic stenosis was found in 12 patients (1.6%), moderate or more aortic regurgitation in 19 patients (2.8%), and moderate or more tricuspid regurgitation in 112 patients (15%). A concomitant diagnosis of coronary artery disease existed in 102 patients (13.4%), and of atrial fibrillation in 177 patients (23.3%). Twelve patients (1.6%) had a dilated ascending aorta. Table 2 provides an overview of mitral valve repair details in the unmatched sternotomy and mini-thoracotomy groups.

**Table 1:** Baseline characteristics of all MVP patients undergoing MVr between 2013 and 2021.

	Total cohort
	*n* = 761
Age	64 (55–72)
Female sex	245 (32.2)
BMI	25.9 (23.3–28.8)
Arterial hypertension	438 (57.6)
Peripheral arterial disease	16 (2.1)
Diabetes	43 (5.7)
COPD	47 (6.2)
Previous stroke	27 (52.9)
Preoperative dialysis	1 (0.1)
Preoperative LVEF	65 (60–69)
Barlow's valve	108 (14.2)
Mitral annular calcification	38 (5)
STS PROM	0.56 (0.31–1.29)
Cardiogenic shock	5 (0.7)
**Echocardiography variables**
Mild aortic stenosis	12 (1.6)
Mild AR	109 (16.2)
Moderate AR	18 (2.7)
Severe AR	1 (0.1)
Mild MS	12 (0.8)
Moderate MR	37 (4.9)
Severe MR	720 (94.9)
Mild TR	266 (35.6)
Moderate TR	101 (13.5)
Severe TR	11 (1.5)
Mild PR	77 (10.9)
Moderate PR	9 (1.3)
Atrial fibrillation	177 (23.3)
Coronary artery disease	102 (13.4)
Ascending aortic dilation	12 (1.6)
Status post mediastinal rediation	1 (0.1)
Dextrocardia	1 (0.1)
HOCM	1 (0.1)

COPD, chronic-obstructive pulmonary disease; LVEF, left ventricular ejection fraction; SMD, standardized mean differences; STS PROM, predicted risk of mortality by STS risk score model. Continuous data presented as median with interquartile range; categorical data presented as numbers with percentages.

**Table 2:** Overall surgical specifics and repair success of MVr regardless of concomitant procedures.

	Total cohort	Sternotomy	Mini-thoracotomy	*p*-value
	*n* = 761	*n* = 498	*n* = 263	
Conversion to full sternotomy	–	–	3 (1.1)	–
**Repair techniques**
Neochords + annuloplasty	433 (56.9)	216 (43.4)	217 (82.5)	**<0**.**001**
Resection + annuloplasty	208 (27.3)	195 (39.2)	13 (4.9)	**<0**.**001**
Neochords + resection + annuloplasty	79 (10.4)	50 (10)	29 (11.0)	0.8
Commissuroplasty + annuloplasty	8 (13.1)	8 (1.6)	0 (0)	0.09
Isolated annuloplasty	10 (1.3)	9 (1.8)	1 (0.4)	0.2
Extensive MAC debridement with posterior annular reconstruction	11 (1.4)	11 (1.4)	0 (0)	**0**.**035**
**Successful repairs**	745 (97.9)	482 (96.8)	263 (100)	**0**.**007**
*Of those, annuloplasty devices implanted*
*Physio I ring*	*632* (*84.8)*	375 (75.3)	261 (99.2)	**<0**.**001**
*Annuloflex band*	*89* (*11.9)*	87 (17.5)	2 (0.8)	**<0**.**001**
*Annuloflex ring*	*12* (*1.6)*	12 (2.4)	0 (0)	**0**.**03**
*Carpentier band*	*2* (*0.3)*	2 (0.4)	0 (0)	0.8
*Carbomedics ring*	*9* (*1.2)*	9 (1.8)	0 (0)	0.07
*Other ring or band*	*1* (*0.1)*	1 (0.2)	0 (0)	1
More than trace residual mitral regurgitation	43 (5.7)	32 (6.4)	11 (4.2)	0.3
**Converted to replacement after failed repair**	16 (2.1)	16 (3.2)	0 (0)	**0**.**007**
*Of those, valve prostheses implanted*
*mechanical valve*	*3* (*18.8)*	*3* (*18.8)*	–	–
*porcine tissue valve*	*13* (*81.3)*	*13* (*81.3)*	–	–

MAC, mitral annular calcification. Data presented as numbers with percentages and compared by Pearson's chi-squared test. Bold *p*-values are <0.05 indicating statistical significance.

### Concomitant procedures

As expected, patients of the sternotomy group underwent significantly more concomitant procedures, as depicted in Supplementary Table S1. Briefly, in the sternotomy group, 98 patients (19.7%) underwent concomitant CABG, 5 patients (1%) aortic valve repair or replacement, and 8 patients (1.6%) ascending aortic replacement with or without root replacement. Regarding concomitant procedures that are feasible *via* sternotomy as well as *via* a mini-thoracotomy approach, LAA occlusion was performed more frequently in the sternotomy group (228 patients (45.8%) vs. 10 patients (3.8%) in the mini-thoracotomy group, *p* < 0.001), as well as the bi-atrial Cox-Maze procedure (85 patients (17.1%) vs. 2 patients (0.8%), *p* < 0.001). The left Cox-Maze procedure was performed in similar proportions in both groups (37 patients (7.4%) in the sternotomy group, 15 patients (5.7%) in the mini-thoracotomy group, *p* = 0.5). A PFO was closed in 66 patients of the sternotomy group (13.3%) and 41 patients of the mini-thoracotomy group (15.6%; *p* = 0.4).

### Changes in institutional volumes and success rates

**Figure 3:**
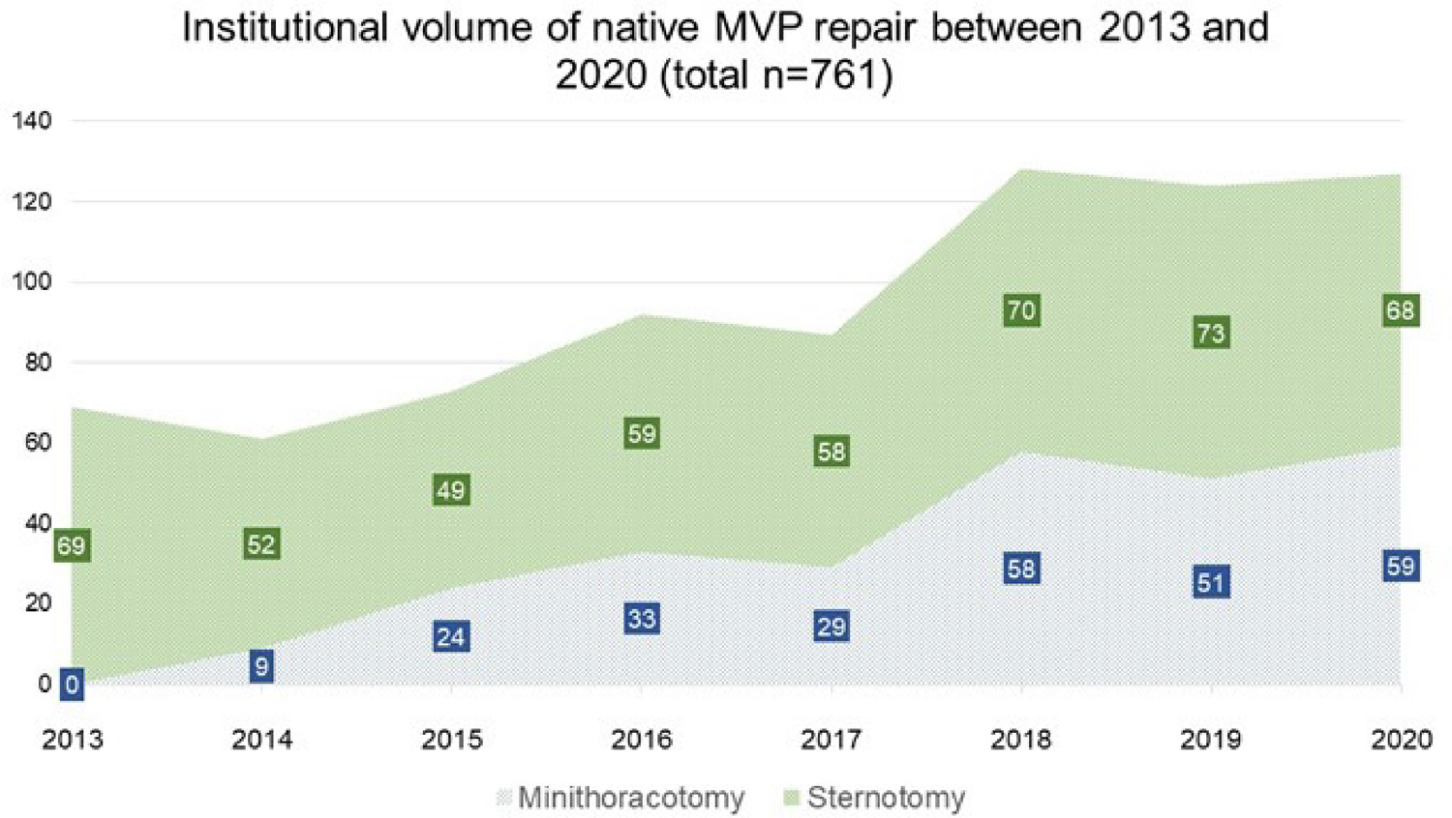
Development of institutional volumes after introduction of minimally invasive MVr program. MVP: Mitral Valve Prolapse.

**Figure 4:**
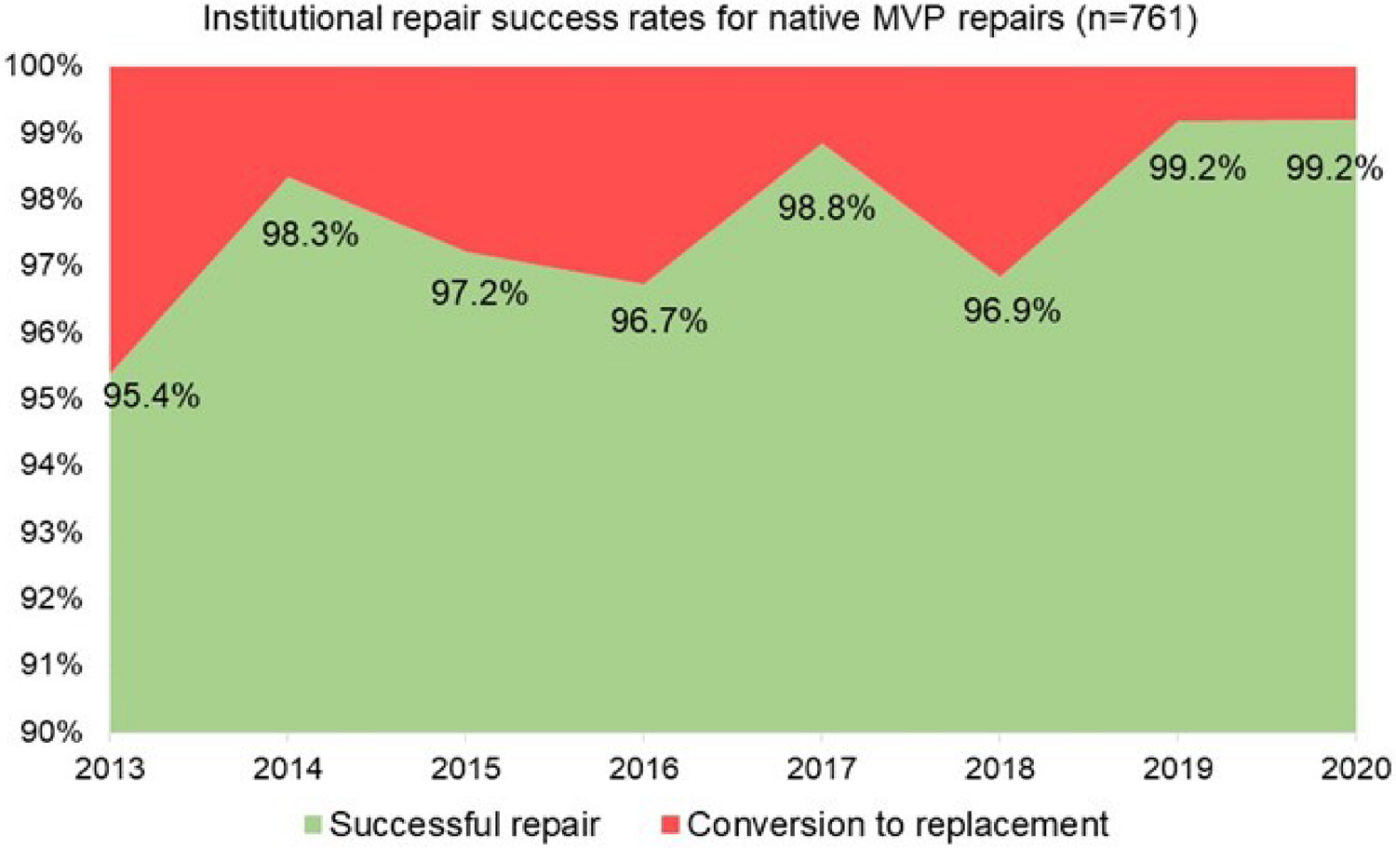
Development of institutional repair success rates for native mitral valve prolapse (MVP).

**Figure 5:**
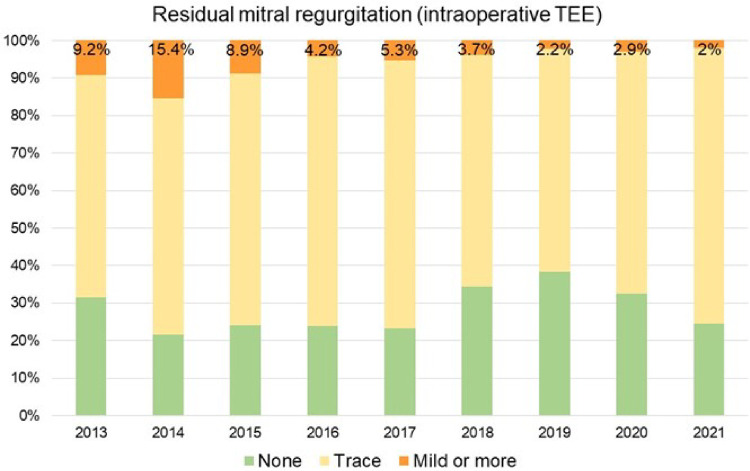
Proportion of patients with mild or more residual mitral regurgitation. TEE: Transesophageal Echocardiography.

We observed a continuous growth in the overall institutional volume of surgery for MVP with increasing proportions of minimally invasive operations since 2014, when the mini-thoracotomy approach was first introduced at our institution (Figure 3). Overall native MVP case volume was *n* = 69 in 2013 and *n* = 127 in 2020. With the start of our dedicated minimally invasive MVr program, institutional repair success rates improved markedly over the years included in this analysis, with 95.4% in 2013 (before the introduction of minimally invasive MVr) vs. 99.2% in 2020 (Figure 4). Residual MR was mild or more in 9.2% of patients in 2013 vs. 2% in 2020, with a continuous reduction over the years (Figure 5).

### Changes in case complexity and surgical techniques

**Figure 6:**
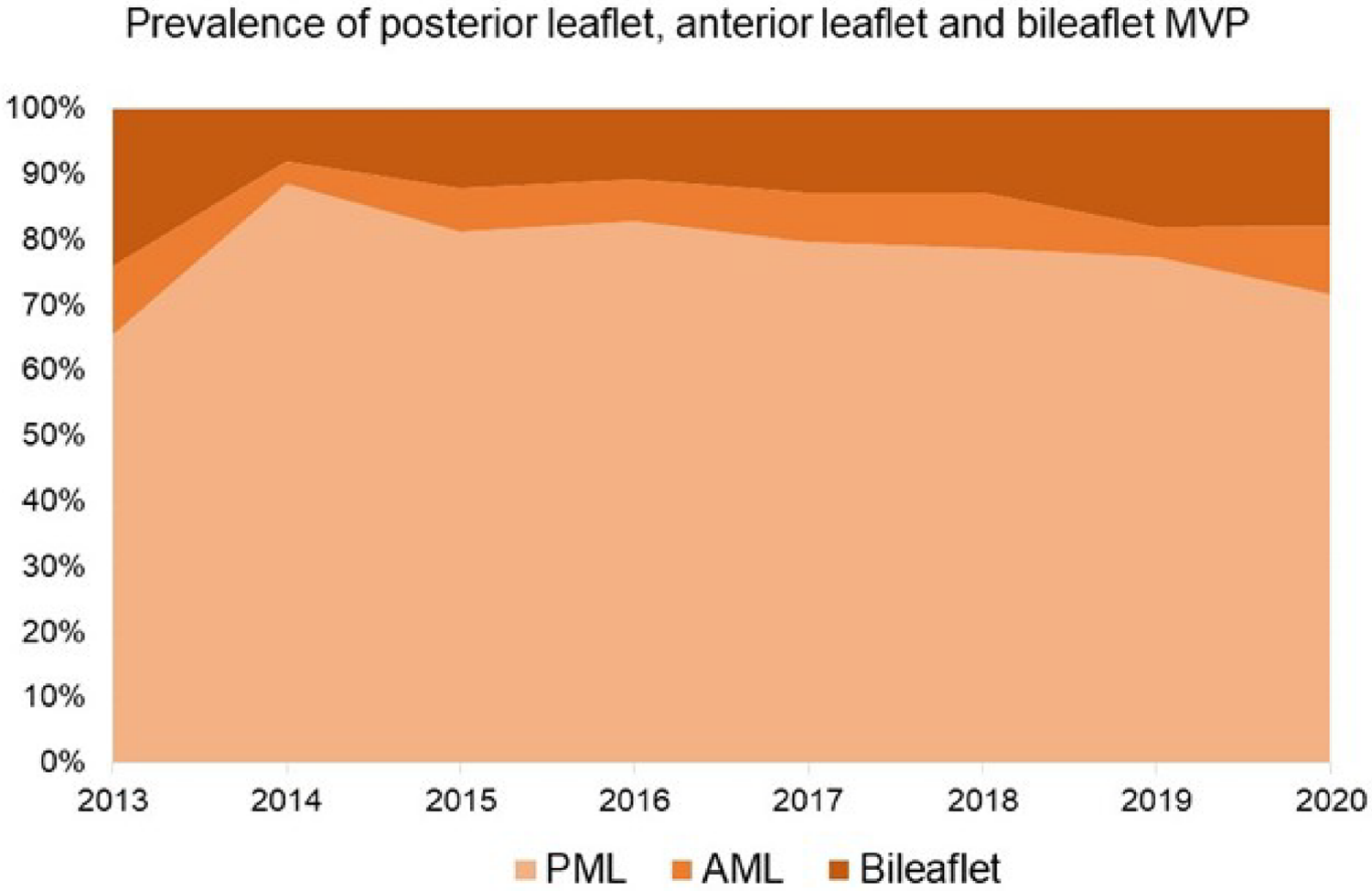
Proportions of patients with posterior leaflet, anterior leaflet, and bileaflet prolapse. AML: Anterior Mitral Leaflet; PML: Posterior Mitral Leaflet. MVP: Mitral Valve Prolapse.

**Figure 7:**
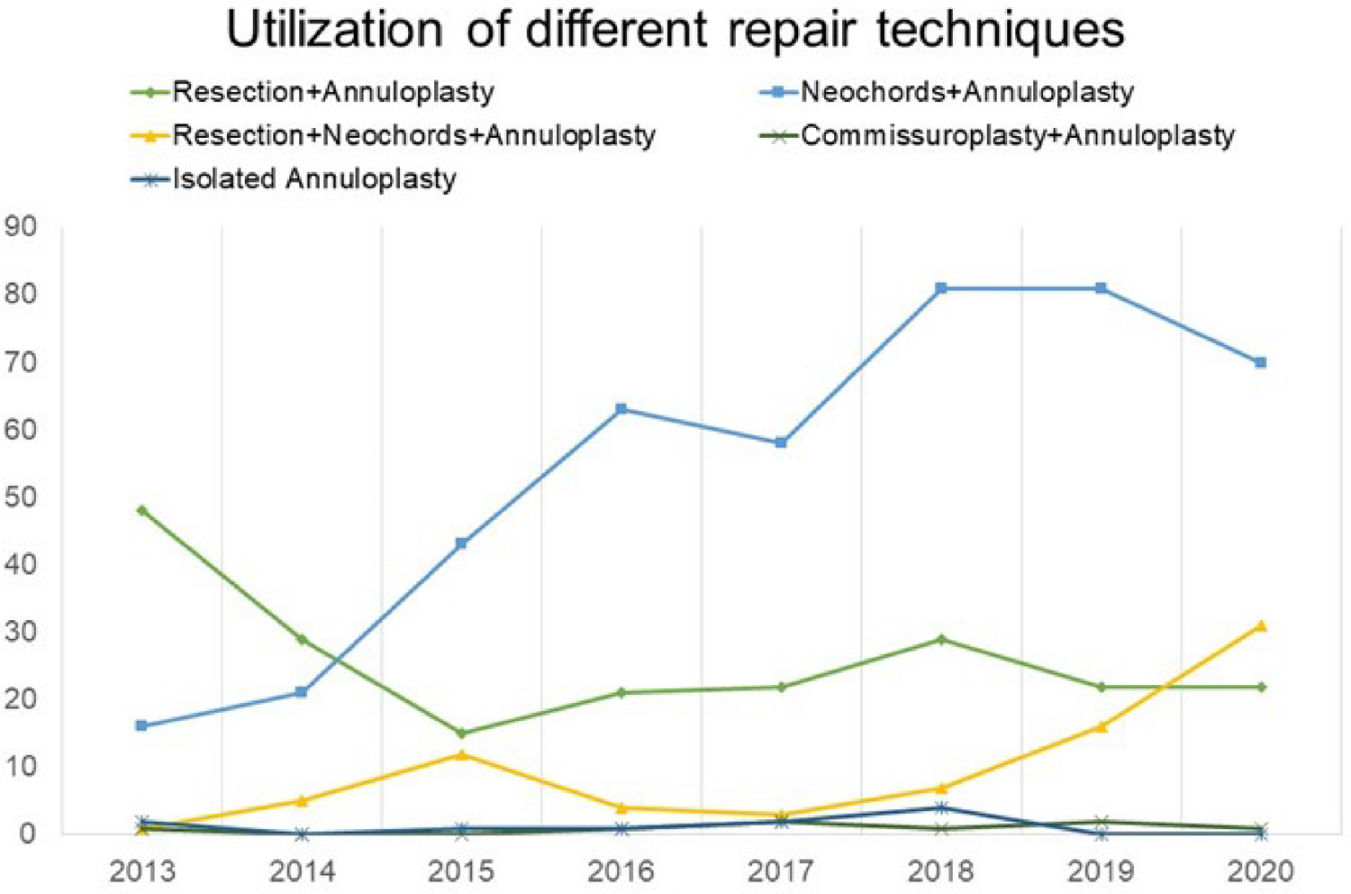
Development of repair techniques over the years.

Since the start of our minimally invasive MVr program, there has been a continuous growth in the proportion of surgically more complex patients [i.e., anterior mitral leaflet (AML) or bileaflet prolapse], with proportions similar to before 2014 (Figure 6). In addition, a steady increase in the utilization of chordal reconstruction with GoreTex neochords and a combination of limited leaflet resection and neochord insertion could be observed. At the same time, a purely resectional technique was utilized less commonly in recent years (Figure 7).

### Outcomes of minimally invasive mitral valve repair

The repair success rate with the minimally invasive approach was 100%, and only 3/263 patients (1.1%) required intraoperative conversion to full sternotomy. Reasons for conversion were bleeding from the aortic root in 2 and an intraoperative localized aortic dissection at the insertion point of the cardioplegia cannula in 1 patient. All 3 patients were discharged home with no significant increase in their hospital stay or further complications.

**Figure 8:**
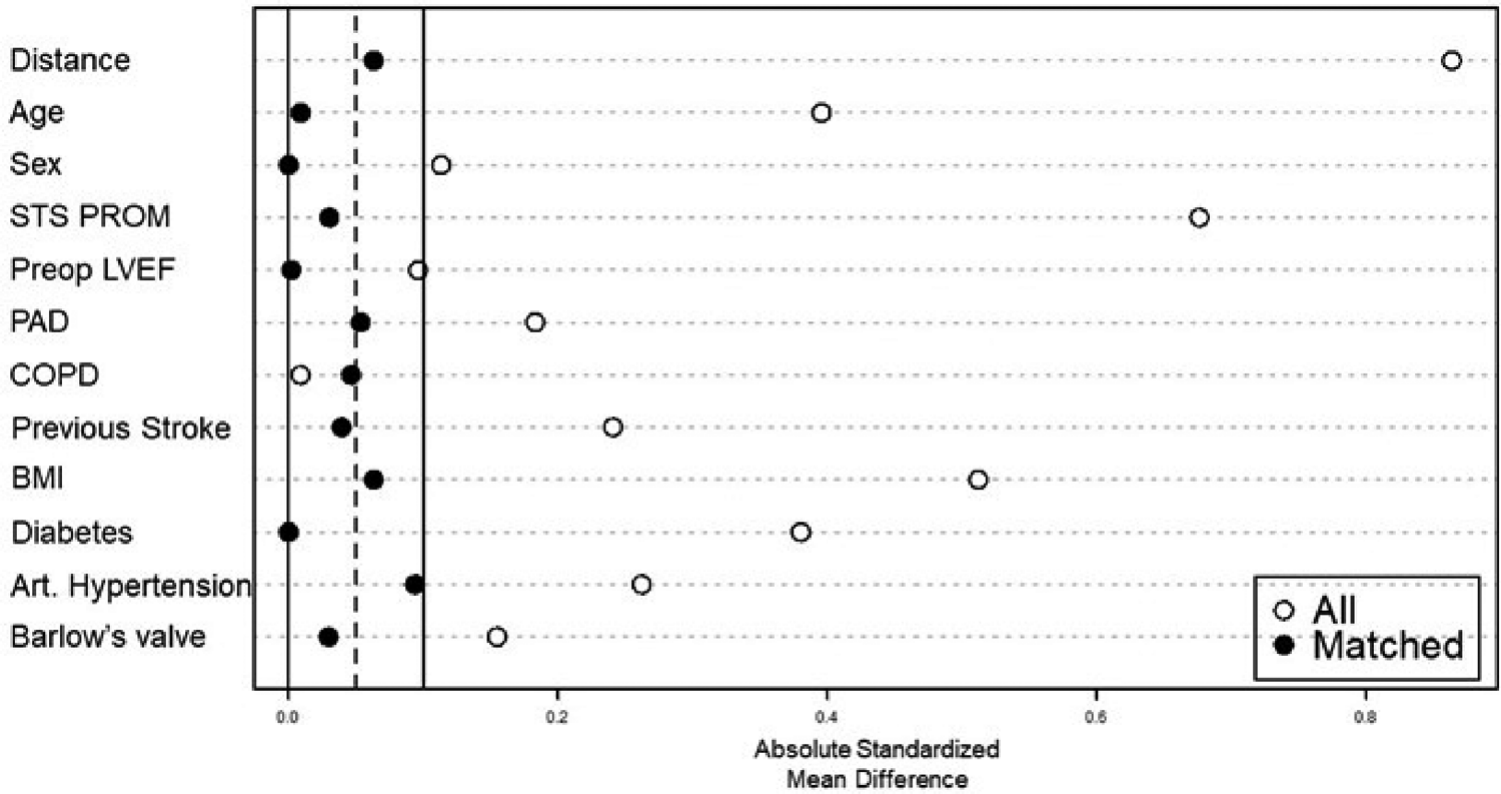
Balancing of covariates by propensity score matching. STS PROM: society of thoracic surgeons–predicted risk of mortality; LVEF: left ventricular ejection fraction; PAD: peripheral artery disease: COPD: chronic obstructive pulmonary disease; BMI: body mass Index.

*For the subgroup analysis of isolated MVr via sternotomy vs.* mini-thoracotomy, we identified 599 patients that received MVr without major concomitant procedures (Figure 1). Of those, 346 were operated *via* sternotomy and 261 *via* right anterolateral mini-thoracotomy. Propensity matching resulted in 214 matched pairs with comparable baseline characteristics (Table 3 and Figure 8). Outcomes of the unmatched groups are summarized in Supplementary Table S2. Between the matched groups, there were significant differences in repair techniques: The neochords + annuloplasty technique was used more frequently in the mini-thoracotomy group (81.8% vs. 38.3% in the sternotomy group, OR 2.1, 95%-CI 1.6–2.8, *p* < 0.001) while the resection + annuloplasty technique was used less often (4.7% vs. 43.5% in the sternotomy group, OR 0.1, 95%-CI 0.05–0.2, *p* < 0.001). Occlusion of the left atrial appendage was also done less frequently in the mini-thoracotomy group (3.3% vs. 40.7% in the sternotomy group, OR 0.09, 95%-CI 0.04–0.2, *p* < 0.001) as well as the bi-atrial Cox-Maze procedure (0.9% vs. 11.7% in the sternotomy group, OR 0.08, 95%-CI 0.009–0.3, *p* = 0.01). There were no significant differences in the rates of PFO closure or the left Cox-Maze procedure. One patient in the sternotomy group required emergency CABG to the circumflex artery, and 1 patient each of the sternotomy and minithoracotomy groups required emergency replacement of the ascending aorta because of an intraoperative localized aortic dissection. Patients operated *via* minithoracotomy had significantly increased cardiopulmonary bypass times of 161 min (IQR 147–185) vs. 116 (98–145, *p* < 0.001); and increased aortic cross-clamp times of 94 min (84–113.8) vs. 88 (70–109, *p* = 0.001). Information on operative details between the matched groups is displayed in Table 4. MVr was successful in 210 patients (98.1%) of the sternotomy and 214 patients (100%) of the mini-thoracotomy group (*p* = 0.9), with no significant differences in residual MR and mean mitral valve pressure gradient (see Table 5).

**Table 3:** Baseline characteristics of the isolated MVr groups before and after propensity score matching.

	Unmatched					Matched				
	Total	Full Sternotomy	Mini- thoracotomy	*p*-value	SMD	Total	Full Sternotomy	Mini- thoracotomy	*p*-value	SMD
	*n* = 599	*n* = 338	*n* = 261			*n* = 428	*n* = 214	*n* = 214		
Age	62 (53.5–69)	59 (52–67)	64 (56–71.3)	**<0**.**001**	0.392	60 (52–68)	60 (53–68)	61 (52–68)	0.7	0.009
Female Sex	206 (34.4)	124 (36.7)	82 (31.4)	0.2	0.111	138 (32.2)	69 (32.2)	69 (32.2)	1	0
Body Mass Index	25.8 (23.3–28.6)	26.4 (23.3–29.5)	25.4 (23.3–27.1)	**<0**.**001**	0.375	25.4 (23.1–27.8)	25.2 (22.7–28.6)	25.6 (23.5–27.3)	0.9	0.051
Arterial Hypertension	322 (53.8)	201 (59.5)	121 (46.4)	**0**.**002**	0.265	214 (50)	112 (52.3)	101 (47.2)	0.5	0.094
Peripheral arterial disease	10 (1.7)	8 (2.4)	2 (0.8)	0.2	0.129	3 (0.7)	2 (0.9)	1 (0.5)	1	0.056
Diabetes	25 (4.2)	11 (6.2)	4 (1.5)	**0**.**008**	0.244	8 (1.9)	4 (1.9)	4 (1.9)	1	0
COPD	29 (4.8)	18 (5.3)	11 (4.2)	0.7	0.052	19 (4.4)	11 (5.1)	8 (3.7)	0.6	0.068
Previous Stroke	30 (5.0)	24 (7.1)	6 (2.3)	**0**.**01**	0.228	9 (2.1)	5 (2.3)	4 (1.9)	1	0.033
Preoperative LVEF	65 (60–70)	65 (60–70)	65 (60-69)	0.3	0.081	65 (60–70)	65 (60–70)	65 (60–69)	0.5	0.002
Barlow's valve	83 (13.9)	54 (16)	29 (11.1)	0.1	0.143	60 (14)	31 (14.5)	29 (13.6)	0.9	0.027
STS PROM	0.46 (0.28–0.9)	0.6 (0.33–1.17)	0.36 (0.25–0.65)	**0**.**002**	0.264	0.41 (0.26–0.72)	0.43 (0.28–0.78)	0.37 (0.25–0.67)	0.1	0.028

COPD, chronic-obstructive pulmonary disease; LVEF, left ventricular ejection fraction; SMD, standardized mean differences; STS PROM, predicted risk of mortality by STS risk score model. Continuous data presented as median with interquartile range; categorical data presented as numbers with percentages. Unmatched comparisons with Wilcoxon rank-sum test and Pearson's chi-squared test; matched comparisons with Wilcoxon sign rank test and McNemar's test. Bold *p*-values indicate statistical significance with an alpha-level of 0.05.

**Table 4:** Surgical details of the matched isolated MVr groups.

	Overall	Sternotomy	Minithoracotomy	OR	95% CI	*p*-value
	*n* = 428	*n* = 214	*n* = 214			
Conversion to sternotomy	2 (1.0)	–	2 (1.0)	–	–	–
**Repair techniques**
Neochords + annuloplasty	257 (60.0)	82 (38.3)	175 (81.8)	2.1	1.6–2.8	**<0**.**001**
Resection + annuloplasty	103 (24.1)	93 (43.5)	10 (4.7)	0.1	0.05–0.2	**<0**.**001**
Resection + neochords + annuloplasty	52 (12.1)	27 (12.6)	25 (11.7)	0.9	0.5–1.7	0.9
Isolated annuloplasty	2 (0.5)	1 (0.5)	1 (0.5)	1	0.01–78	1
Limited MAC debridement	2 (0.5)	2 (0.9)	0 (0)	0	0–5.3	0.5
**Concomitant procedures**
LAA occlusion	94 (19.9)	77 (40.7)	7 (3.3)	0.09	0.04–0.2	**<0**.**001**
Biatrial Cox-Maze	27 (6.3)	25 (11.7)	2 (0.9)	0.08	0.009–0.3	**0**.**01**
Left atrial Cox-Maze	26 (6.1)	14 (6.5)	12 (5.6)	0.9	0.4–2	0.8
PVI	1 (0.2)	1 (0.5)	0 (0.0)	0	0–39	1
PFO closure	61 (14.3)	28 (13.1)	33 (15.4)	1.2	0.7–2	0.6
ASD closure	1 (0.2)	1 (0.5)	0 (0.0)	0	0–39	1
**Emergency procedures**
Emergency CABG	1 (0.2)	1 (0.5)	0 (0.0)	0	0–39	1
Emergency Ascending replacement	2 (0.5)	1 (0.5)	1 (0.5)	1	0.01–78	1
**Successful repair**	424 (99.1)	210 (98.1)	214 (100)	1.02	0.8–1.2	0.9
Physio I ring	367 (85.7)	155 (73.8)	212 (99.1)	1.4	1.1–1.7	**0**.**003**
Annuloflex band	45 (10.5)	43 (20.5)	2 (0.9)	0.05	0.005–0.2	**<0**.**001**
Annuloflex ring	7 (1.6)	7 (3.3)	0 (0.0)	0	0–0.7	0.02
Carpentier band	1 (0.2)	1 (0.5)	0 (0.0)	0	0–39	1
Carbomedics ring	3 (0.7)	3 (1.4)	0 (0.0)	0	0–2.4	0.3
**Conversion to replacement**	4 (0.9)	4 (1.9)	0 (0)	0	0–1.5	0.1
Porcine tissue valve	4 (100)	4 (100)	–	–	–	–
Cross-clamp time (min)	92.5 (79–111.3)	88 (70–109)	94 (84–113.8)	–	–	**0**.**001**
Cardiopulmonary bypass time (min)	147 (115–170)	116 (98–145.3)	161 (147.3–185)	–	–	**<0**.**001**

ASD, atrial septal defect; CABG, coronary artery bypass grafting; LAA, left atrial appendage; MAC, mitral annular calcification; PFO, persistent foramen ovale; PVI, pulmonary vein isolation. Continuous data presented as median with interquartile range and compared by Wilcoxon signed rank test; Categorical data presented as numbers with percentages as well as odds ratios with 95% confidence intervals, compared by McNemar's test. Bold *p*-values indicate statistical significance with an alpha-level of 0.05.

**Table 5:** Echocardiographic assessment of repair results in the matched isolated MVr groups.

	Matched groups			
	Overall	Sternotomy	Minithoracotomy	
	*n* = 428	*n* = 214	*n* = 214	*p*-value
**Residual mitral regurgitation**				0.3
None	130 (30.6)	57 (26.9)	73 (34.3)	
Trace	274 (64.5)	143 (67.5)	131 (61.5)	
Mild	20 (4.7)	11 (5.2)	9 (4.2)	
Moderate	1 (0.2)	1 (0.5)	0 (0)	
**Mean trans-mitral pressure gradient**	3 (2;4)	3 (2;3)	3 (2;4)	0.2

Data presented as numbers with percentages and compared by Pearson's chi-squared test or median (interquartile range) and compared using Wilcoxon rank-sum test.

Patients operated *via* mini-thoracotomy had a slightly higher postoperative LVEF than those operated *via* sternotomy (62% [57–66] vs. 62% [55–66], *p* = 0.03), required significantly shorter time on mechanical ventilation (4.4 h [2.4–6.3] vs. 4.8 h [3.3–7.5], *p* = 0.002) and were hospitalized significantly shorter (5 days [4–5] vs. 6 days [5–7], *p* < 0.001). There were no significant differences between the matched groups regarding postoperative complications. Operative mortality was 0% in both groups, while 30-day mortality was 0.9% in the sternotomy and 0% in the minithoracotomy group (*p* = 0.5). Kaplan-Meier method with log-rank test revealed no difference regarding long-term freedom from re-operation and death (*p* = 0.19, Supplementary Figure S1). Postoperative outcomes of the matched groups are summarized in Table 6.

**Table 6:** Postoperative outcomes of the matched isolated MVr groups.

Matched groups						
	Overall	Sternotomy	Minithoracotomy	OR	95% CI	*p*-value
	*n* = 428	*n* = 214	*n* = 214			
Total ventilation time (h)	4.6 (3–6.7)	4.8 (3.3–7.5)	4.4 (2.4–6.3)	–	–	**0.002**
Re-intubation	4 (0.9)	1 (0.5)	3 (1.4)	3	0.2–157	0.6
Blood transfusion	51 (11.9)	28 (13.1)	23 (10.7)	0.8	0.5–1.5	0.6
Intensive care unit stay (h)	25 (22.7–41)	26 (23–42)	25 (22.5–33.4)	–	–	0.4
Postop LVEF (%)	62 (56–66)	62 (55.3–66)	62 (57–66)	–	–	**0.03**
Re-exploration for bleeding	11 (2.6)	4 (1.9)	7 (3.3)	1.8	0.4–8.2	0.5
Re-intervention for valve	1 (0.2)	1 (0.5)	0 (0)	0	0–39	1
Re-exploration for other reasons	1 (0.2)	1 (0.5)	0 (0)	0	0–39	1
Sternal dehiscence	1 (0.2)	1 (0.5)	–	–	–	-
Stroke	1 (0.2)	1 (0.5)	0 (0.0)	0	0–39	1
Extended ventilation	10 (2.3)	6 (2.8)	4 (1.9)	0.7	0.1–2.8	0.8
Tracheostomy	0 (0)	0 (0)	0 (0)	–	–	–
Pneumonia	2 (0.5)	2 (0.9)	0 (0)	0	0–5.3	0.5
Pleural effusion requiring intervention	11 (2.6)	9 (4.2)	2 (0.9)	0.2	0.02–1.07	0.07
Pneumothorax	4 (0.9)	2 (0.9)	2 (0.9)	1	0.07–13.8	1
Renal failure	1 (0.2)	1 (0.5)	0 (0)	0	0–39	1
Dialysis	0 (0)	0 (0)	0 (0)	–	–	–
New permanent pacemaker	6 (1.4)	5 (2.3)	1 (0.5)	0.2	0.004–1.8	0.2
Atrial fibrillation	61 (28.3)	60 (28)	61 (28.5)	1.02	0.7–1.5	1
Cardiac tamponade	1 (0.2)	1 (0.5)	0 (0.0)	0	0–39	1
30-day mortality	2 (0.5)	2 (0.9)	0 (0)	0	0–5.3	0.5
In-house mortality	0 (0)	0 (0)	0 (0)	–	–	–
Hospital length of stay	5 (4–6)	6 (5–7)	5 (4–5)	–	–	**<0.001**

CI, confidence interval; LVEF, left ventricular ejection fraction; OR, odds ratio. Continuous data presented as median with interquartile range and compared by Wilcoxon signed rank test; Categorical data presented as numbers with percentages as well as odds ratios with 95% confidence intervals, compared by McNemar's test. Bold *p*-values indicate statistical significance with an alpha-level of 0.05.

### Initial results with the robotic approach

We included all patients (*n* = 16) that underwent robotically assisted MVr at our institution. Repair success rate was 100%, with one patient requiring a second CPB run for residual mild-moderate MR after the initial repair attempt. Repair techniques included resection + annuloplasty, neochord implantation + annuloplasty as well as the combined technique. Three patients had no residual MR and 13 had trace residual MR after robotic MVr. Concomitant PFO closure was done in 4 patients (25%). Mean CPB and aortic cross-clamp times were 239 ± 59 min and 153 ± 48 min respectively, which is substantially longer than in the matched minimally invasive and full sternotomy groups of isolated MVr. Mean hospitalization time was 5.4 ± 1.8 days and there were no relevant postoperative complications during the hospitalization. Preoperative characteristics and results of the robotic cohort are displayed in Table 7.

**Table 7:** Preoperative characteristics, surgical details, and outcomes of the first 16 robotic MVr patients.

	Total robotic cohort (*n* = 16)
Age	57.3 (11.2)
Female sex	4 (25)
Body mass index	25.4 (3.1)
Arterial hypertension	4 (25)
Diabetes	0 (0)
Previous stroke	0 (0)
Peripheral vascular disease	1 (6.3)
Coronary artery disease not requiring intervention	3 (18.8)
Chronic obstructive pulmonary disease	1 (6.3)
Left ventricular ejection fraction	61.8 (3.3)
STS PROM	0.4 (0.4)
**Mitral valve pathology**
Posterior leaflet prolapse	14 (87.5)
Bileaflet prolapse	2 (12.5)
**Repair techniques**
Neochords + annuloplasty	11 (68.8)
Resection + annuloplasty	3 (18.8)
Resection + neochords = annuloplasty	2 (12.5)
Concomitant PFO closure	4 (25)
Second CPB run necessary	1 (6.3)
Perfusion time	239 (59)
Cross-clamp time	153 (48)
**Residual Mitral regurgitation**
None	3 (18.8)
Trace	13 (81.3)
Mean pressure gradient post repair	2 (0.65)
Hospital length of stay	5.4 (1.8)

CPB, cardiopulmonary bypass; PFO, persistent foramen ovale; STS PROM, society of thoracic surgeons predicted risk of mortality.

Continuous variables presented as mean with standard deviation, categorical variables as numbers with percentages.

## Discussion

### Minimally invasive mitral valve repair

Since the first reported case of minimally invasive mitral surgery in 1996 (10), minimally invasive MVr has undergone a significant and continuous expansion (11), supported by large series demonstrating the safety and benefit of this type of procedure (3–6). However, full sternotomy remains the most used approach for MVr. In this retrospective analysis, we observed that setting up a program dedicated to the incorporation of mini-thoracotomy for MVr has made it possible to change the management standard in an efficient and controlled manner without significant increases in complication rates. As shown by many groups, the benefits of minimally invasive MVr can be shorter postoperative ventilation times and shorter overall hospital stay without increased postoperative complications, and at similar procedural costs compared to the sternotomy approach (12–15). Our results are in line with these previous studies, showing significantly decreased postoperative ventilation time and hospital length of stay. A possible reason for shorter ventilation time, which we were not able to explore based on the presented data, may be less postoperative pain due to the smaller incision and less intraoperative spreading with quicker recovery to sufficient breathing patterns triggering earlier extubation. Another important benefit of the minimally invasive approach lies in an expedited recovery of these patients from an open-heart surgery. Without sternotomy-related precautions with regards to mobility, these otherwise healthy adults enjoy an earlier resumption of their active lifestyle as well as an earlier return to their work environment.

An important observation was the increase in overall MVr volume at our institution after starting the mini-thoracotomy program. This resulted in regular interaction of a team of experienced MV surgeons and echocardiographers for preoperative case planning and intraoperative consultation which we believe significantly contributes to patient safety and improved repair success rates (Figures 4, 5). This is in line with several prior reports that detected a strong relationship between individual surgeon and institutional case volumes with repair success rates. We believe that this benefit has a wider beneficial effect as well. Chikwe et al. found that even surgeons with lower MVr volumes (<25/year) have higher repair success rates when operating at the same institutions as higher-volume surgeons (>50/year) when compared to working at other institutions. Newell et al. also recently showed that at an institution with high MVr volumes, outcomes of low- and high-volume surgeons were comparable (16–19). Higher volumes also improve educational experiences with regular trainee exposure to complex MVr, allowing for stepwise teaching opportunities.

It should be emphasized that the quality of MVr was not negatively affected by choosing a minimally invasive approach, with a repair success rate of 100% in this cohort and no differences in residual MR compared to patients operated *via* sternotomy. In addition, neochord insertion, which might have superior long-term success rates (1, 20, 21), was more frequently used in mini-thoracotomy patients of our cohort. Longer lines of coaptation found after the neochord technique may translate into improved long-term outcomes (22, 23). As displayed in Figure 7, increasing expertise in complex MVr *via* mini-thoracotomy has changed our institutional approaches to MVP and a combination of resection and neochord insertion is not uncommon. In our opinion, it is crucial to be able to provide a wide range of repair techniques tailored to individual valve anatomy, without necessarily restricting the approach to either “resecting” or “respecting”.

With growing institutional and individual experience regarding minimally invasive MVr, a growth in proportion of more complex MVP cases was observed at MGH, such as anterior leaflet and bileaflet prolapse, undergoing MVr (Figure 6). While isolated posterior leaflet prolapse is the predominant lesion in MVP and associated with excellent repair rates, anterior and bileaflet prolapse present a more challenging surgical task. Yet, repairing these pathologies is feasible with similar long-term outcomes as MVr for isolated posterior leaflet prolapse and should be the primary goal for treatment of MVP (20, 24).

### Toward the utilization of robotic support for minimally invasive MVr

Especially in recent years, cardiac surgeons are confronted with the utilization of robotic support within a growing range of surgical specialties, sparking interest of patients and referring cardiologists. Perceived as an advance of established minimally invasive procedure, introducing robotically assisted cardiac surgery faces similar challenges as surgical training in general (9, 25). Robotic MV surgery was first reported on in 1998 (10, 26), with subsequent encouraging multicenter studies on the success of robotic MVr (27). However, robotic deployment, in addition to the difficulties inherent to a new procedure, is even more limited by its cost and low availability of equipment. Nevertheless, the learning curves appear to be acceptable, with reported rapid reductions in cross-clamp times and stabilization after 20–30 cases (28, 29). As mentioned above, facilitation of papillary muscle and mitral valve exposure and free multi-dimensional movement of surgical instruments are advantages of robotic MV surgery compared to the established methods.

At MGH, a stepwise approach is now followed to shift from standard minimally invasive to robotic MVr. Badhwar et al. proposed that institutional cardiac surgery case volumes be >250 cases/year, that an experienced team of anesthesiologists and perfusionists be present, and that the individual surgeon have at least 15 minimally invasive MVr cases in his or her record, to allow for safe initiation of a robotic MVr program. These criteria were by far exceeded in our case: Institutional yearly volume = ca. 1900 cases with an established team of cardiac anesthesiologists and perfusionists; individual volume of minimally invasive MVr >250 cases. Use of robotic platform for MVr was introduced only when minimally invasive MVr outcomes were excellent and comparable to those of sternotomy MVr, demonstrated by our subgroup analysis. Through guidance of experienced robotic MVr surgeons at all stages of training (theoretical, simulator, and cadaver training) as well as in-person and remote proctoring (Figure 2), we were able to launch a successful robotic MVr program that thus far has yielded satisfying results with a 100% repair success rate. As previously published, a decrease in aortic cross-clamp time can be expected over the next phase of robotic MVr cases. Especially through development of advanced simulation settings, adaptation of robotic surgery can be facilitated for surgeons already competent in complex minimally invasive MVr (30–32).

In an era of the rapid growth of transcatheter techniques, new percutaneous approaches to MVP repair are being proposed (33). However, the possibility of repairing all aspects of MVP (leaflet, annular and subvalvular abnormalities) and especially the availability of durable and safe annuloplasty currently favors surgical MVr. By reducing invasiveness and tissue trauma using minimally invasive and robotic approaches, earlier discharge and reduced use of rehabilitation facilities are possible, with reduced scarring to improve quality of life and patient satisfaction (34, 35).

It is our hope that the very good results of the presented cohort at an institution that fairly recently had started a minimally invasive MVr program might encourage centers to enable experienced surgeons and trainees to gain competence in minimally invasive MVr *via* formal training programs and interinstitutional proctoring support.

### Limitations

This work presents a retrospective analysis limited to a tertiary care center. Although propensity score matching was used to compare outcomes of minimally invasive and conventional MVr, remaining selection bias as well as surgeon bias need to be considered for the evaluation of the presented results. However, careful application of exclusion criteria and matching served to compare contemporary patient cohorts at a large-volume center. Regarding training aspects and institutional program, the surgical team at an institution like ours is large enough to have a reference surgeon in this type of procedure, and the training of residents is well-structured and standardized. The presented results and educational aspects might therefore not be entirely applicable to non-academic and lower volume centers.

## Conclusions

Implementing a minimally invasive mitral surgery approach in the framework of a dedicated support program allows for rapid modification of institutional approaches to complex MVr, with improved institutional outcomes and diversification of repair techniques. Based on an established and successful minimally invasive MVr program, the introduction of robotic support can then be accomplished without compromising patient safety, if a team-based stepwise training protocol is followed. In-person and remote proctoring are key elements for learning new surgical techniques and can be utilized to accomplish a more wide-spread use of less-invasive MVr.

## Data Availability

The data underlying the analysis cannot be shared due to data protection of the included individuals. Further inquiries can be directed to the corresponding author.
