# Supplementary material for: Building a successful minimally invasive mitral valve repair program before introducing the robotic approach: The Massachusetts General Hospital experience
Source: Front Cardiovasc Med. 2023 Mar 21;10:1113908. doi: 10.3389/fcvm.2023.1113908 (PMC10070799; doi:10.3389/fcvm.2023.1113908)
Supplement: Supplementary file 1 [file Table1.docx]

# Supplementary Material

*Supplemental Table 1: Concomitant procedures in all patients with Mitral Valve Prolapse undergoing Mitral Valve Repair**

|  | **Total cohort** | **Sternotomy** | **Minithoracotomy** | **p-value** |
| --- | --- | --- | --- | --- |
|  | **n=761** | **n=498** | **n=263** |  |
| LAA exclusion or amputation | 238 (31.3) | 228 (45.8) | 10 (3.8) | **<0.001** |
| Biatrial Maze | 87 (11.4) | 85 (17.1) | 2 (0.8) | **<0.001** |
| Left Maze | 52 (6.8) | 37 (7.4) | 15 (5.7) | 0.5 |
| PVI | 4 (0.5) | 4 (0.8) | 0 (0) |  |
| CABG | 98 (12.9) | 98 (19.7) | 0 (0) |  |
| TV annuloplasty | 43 (5.7) | 41 (8.2) | 2 (0.8) | **<0.001** |
| Additional TV repair | 10 (1.3) | 10 (2) | 0 (0) | **0.048** |
| AV replacement | 2 (0.3) | 2 (0.4) |  |  |
| AV repair (subcommissural annuloplasty) | 3 (0.4) | 3 (0.6) |  |  |
| AV fibroelastoma removal | 1 (0.1) | 1 (0.2) |  |  |
| AV inspection | 2 (0.3) | 2 (0.4) |  |  |
| Ascending replacement | 7 (0.9) | 7 (1.4) |  |  |
| Valve-sparing root replacement | 1 (0.1) | 1 (0.2) |  |  |
| PFO closure | 107 (14.1) | 66 (13.3) | 41 (15.6) | 0.4 |
| ASD closure | 3 (0.4) | 3 (0.4) |  |  |

AV=aortic valve; ASD=atrial septal defect; CABG=coronary artery bypass grafting; LAA=left atrial appendage; PFO=persistent foramen ovale; PVI=pulmonary vein isolation; TV=tricuspid valve

*Those not usually feasible via minithoracotomy were not compared statistically. Data presented as numbers with percentages with odds ratios as applicable; were compared using Fisher’s exact test. Bold p-values are <0.05 indicating statistical significance.

*Supplemental Table 2: Outcomes of the unmatched isolated Mitral Valve repair groups*

| **Unmatched groups** | | | | | | |
| --- | --- | --- | --- | --- | --- | --- |
|  | **Overall** | **Sternotomy** | **Mini-thoracotomy** | **OR** | **95% CI** | **p-value** |
|  | **n=599** | **n=338** | **n=261** |  |  |  |
| Total ventilation time (h) | 4.5 (3-6.8) | 5 (3.4-7.9) | 4 (2-5.9) | - | - | **0.003** |
| Re-intubation | 6 (1) | 3 (0.9) | 3 (1.1) | 1.3 | 0.2-9.8 | 1 |
| Blood transfusion | 66 (14.4) | 60 (17.8) | 26 (10) | 0.5 | 0.3-0.9 | **0.007** |
| ICU stay (h) | 25.6 (23-44) | 26 (23-47.9) | 25 (23-32) | - | - | **0.006** |
| Postop LVEF (%) | 60 (54.8-65.3) | 59.5 (54-64) | 63 (65.3-66) | - | - | 0.06 |
| Re-exploration for bleeding | 13 (2.2) | 5 (1.5) | 8 (3.1) | 2.1 | 0.6-8.3 | 0.3 |
| Re-intervention for valve | 1 (0.5) | 1 (0.8) | 0 (0.0) | - | - | 1 |
| Re-exploration for other reasons | 2 (0.3) | 2 (0.6) | 0 (0) | 0 | 0-6.9 | 0.5 |
| Sternal dehiscence | 1 (0.5) | 1 (0.8) | 0 (0.0) | - | - | 1 |
| Stroke | 4 (0.7) | 3 (0.9) | 1 (0.4) | 0.4 | 0.008-5.4 | 0.6 |
| Extended ventilation | 25 (4.2) | 19 (5.6) | 6 (2.3) | 0.4 | 0.1-1.05 | 0.06 |
| Tracheostomy | 2 (0.3) | 2 (0.6) | 0 (0) | 0 | 0-6.9 | 0.5 |
| Pneumonia | 6 (1) | 5 (1.5) | 1 (0.4) | 0.3 | 0.005-2.3 | 0.2 |
| Pleural effusion requiring intervention | 22 (3.7) | 19 (5.6) | 3 (1.1) | 0.2 | 0.04-0.7) | **0.008** |
| Pneumothorax | 9 (1.5) | 6 (1.8) | 3 (1.1) | 0.6 | 0.1-3 | 0.7 |
| Renal failure | 5 (0.8) | 5 (1.5) | 0 (0) | 0 | 0-1.4 | 0.07 |
| Dialysis | 4 (0.7) | 4 (1.2) | 0 (0) | 0 | 0-2 | 0.1 |
| New PPM | 15 (2.5) | 14 (4.1) | 1 (0.3) | 0.09 | 0.002-0.6 | **0.002** |
| Atrial fibrillation | 166 (27.7) | 94 (27.8) | 72 (27.6) | 1.01 | 0.7-1.5 | 1 |
| Tamponade | 2 (0.3) | 2 (0.6) | 0 (0) | - | - | 0.5 |
| 30-day mortality | 5 (0.8) | 5 (1.5) | 0 (0) | 0 | 0-1.4 | 0.07 |
| In-house mortality | 2 (0.3) | 2 (0.6) | 0 (0) | - | - | 0.5 |
| Hospital length of stay | 5 (4-7) | 6 (5-7) | 5 (4-5) | - | - | **<0.001** |
| Cross-clamp time  (min) | 93 (78-112) | 90.5 (71-112) | 94 (83-111.3) | - | - | **0.002** |
| CPB time (min) | 146 (113-167) | 119 (98-148) | 160 (147-183) | - | - | **<0.001** |
| **Residual Mitral regurgitation** |  |  |  |  |  | 0.09 |
| None | 177 (29.8) | 87 (26.1) | 90 (34.6) |  |  |  |
| Trace | 389 (65.6) | 230 (69.1) | 159 (61.2) |  |  |  |
| Mild | 25 (4.2) | 14 (4.2) | 11 (4.2) |  |  |  |
| Moderate | 2 (0.3) | 2 (0.6) | 0 (0.0) |  |  |  |
| **Mitral valve mean gradient** |  |  |  |  |  | 0.7 |
| 1 | 25 (5.9) | 17 (7.3) | 8 (4.2) |  |  |  |
| 2 | 137 (32.3) | 78 (33.5) | 59 (30.9) |  |  |  |
| 3 | 161 (38.0) | 85 (36.5) | 76 (39.8) |  |  |  |
| 4 | 67 (15.8) | 35 (15.0) | 32 (16.8) |  |  |  |
| 5 | 24 (5.7) | 13 (5.6) | 11 (5.8) |  |  |  |
| 6 | 8 (1.9) | 4 (1.7) | 4 (2.1) |  |  |  |
| 7 | 1 (0.2) | 0 (0.0) | 1 (0.5) |  |  |  |
| 8 | 1 (0.2) | 1 (0.4) | 0 (0.0) |  |  |  |

CI=confidence interval; CPB=cardiopulmonary bypass; ICU=intensive care unit; LVEF=left ventricular ejection fraction; OR=odds ratio; PPM=permanent pacemaker. Continuous data presented as median with interquartile range and compared by Wilcoxon rank-sum test; Categorical data presented as numbers with percentages as well as odds ratios with 95% confidence intervals, compared by Pearson’s chi-squared test or Fisher’s exact test, as appropriate. Bold p-values are <0.05 indicating statistical significance.
