# Supplementary figures and images for: Building a successful minimally invasive mitral valve repair program before introducing the robotic approach: The Massachusetts General Hospital experience
Source: Front Cardiovasc Med. 2023 Mar 21;10:1113908. doi: 10.3389/fcvm.2023.1113908 (PMC10070799; doi:10.3389/fcvm.2023.1113908)

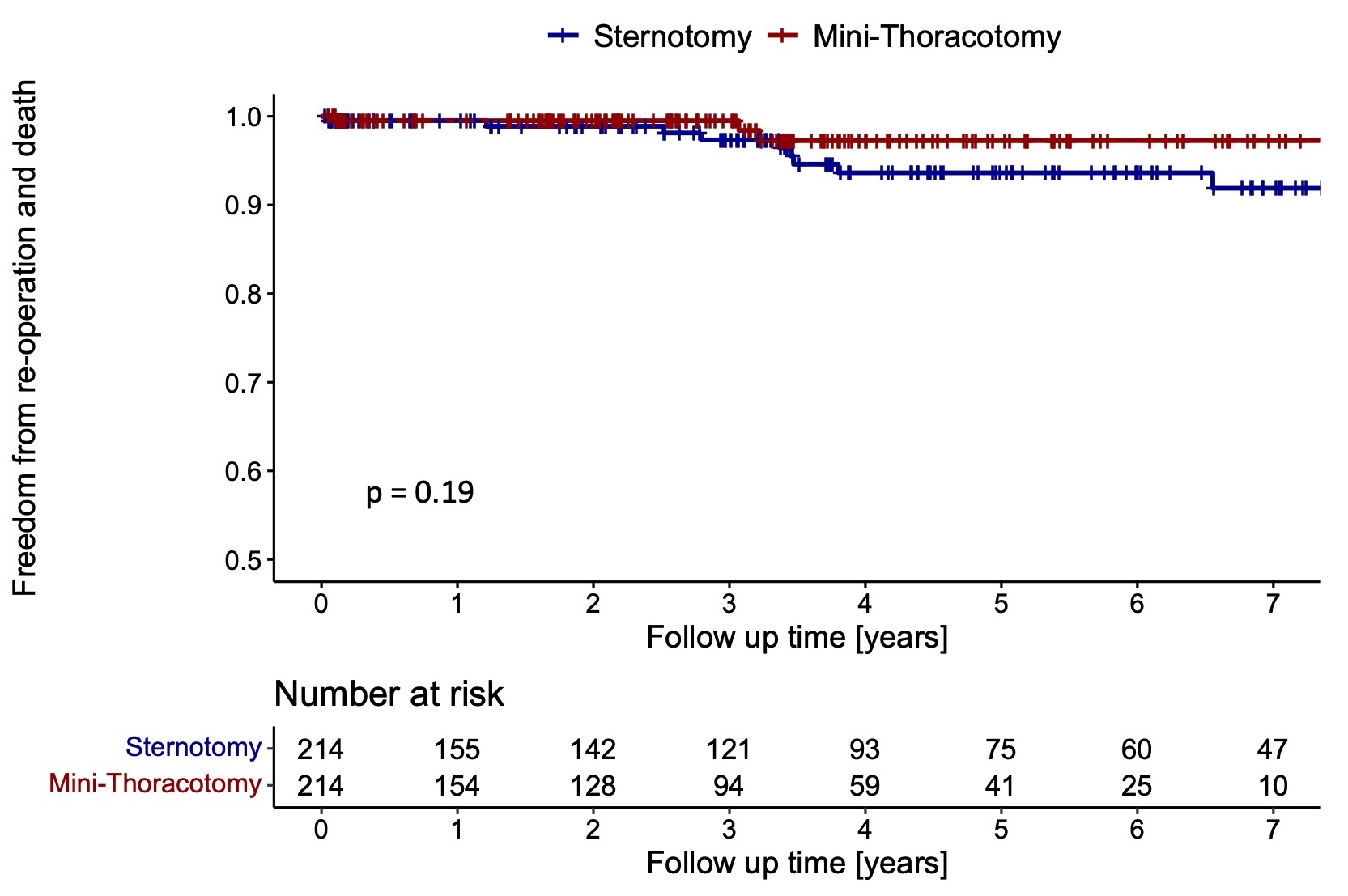

Supplement: Supplementary file 2 [file Image1.jpeg]
